# ReNU Syndrome due to a de novo *RNU4-2* Variant as a Novel Genetic Cause of Proteinuria

**DOI:** 10.1016/j.xkme.2025.101202

**Published:** 2025-12-10

**Authors:** William Morello, Greta Armaroli, Donatella Milani, Anita Sofia Bellotti, Paola Castelli, Elena Cicchetti, Alessandro Del Gobbo, Alessandra De Franco, Giovanni Montini

**Affiliations:** 1 Pediatric Nephrology, Dialysis and Transplant Unit, Fondazione IRCCS Ca' Granda, Ospedale Maggiore Policlinico, Milano, Italy; 2 Fondazione IRCCS Ca' Granda, Ospedale Maggiore Policlinico Milano, Italy; 3 Division of Pathology, Fondazione IRCCS Ca’ Granda, Ospedale Maggiore Policlinico, Milano, Italy; 4 Department of Clinical Sciences and Community Health, Dipartimento di Eccellenza 2023-2027, University of Milan, Milano, Italy

**Keywords:** Proteinuria, ReNU syndrome, RNU4-2, steroid-resistant nephrotic syndrome

## Abstract

Proteinuria has been linked to several genetic disorders, providing valuable insights into its pathophysiology. ReNU syndrome, a recently described condition caused by heterozygous variants in the *RNU4-2* gene, is characterized by intellectual disability, microcephaly, and multisystemic features. Kidney involvement has been reported exclusively as anatomical abnormalities.

Here, we presented a girl with isolated proteinuria and ReNU syndrome. Her prenatal history was notable for a small head circumference and reduced brain hemispheres. She was referred to our clinic at 3 months of age for isolated proteinuria. Physical examination revealed microsomia, strabismus, and dysmorphic features. Kidney ultrasound was unremarkable, and edema was never observed. A kidney biopsy showed minimal change disease with slight podocyte effacement. Treatment with prednisone was ineffective, and antiproteinuric agents were started. At her last follow-up, at age 16 years, nephrotic-range proteinuria persists with normal kidney function. Genetic testing before 2024 yielded no diagnosis, but whole-genome sequencing analysis later identified a de novo variant in the *RNU4-2* gene (n.64_65insT), confirming ReNU syndrome.

This case is the first documented report of isolated, persistent proteinuria in ReNU syndrome. We recommend testing for *RNU4-2* variants in patients with unexplained proteinuria and syndromic features and suggest regular monitoring for proteinuria in individuals with ReNU syndrome.

Advances in genetic research have significantly improved our understanding of the genetic causes of proteinuria over the last years.^1^, ^2^, ^3^ Several gene alterations have now been linked to proteinuria, shedding light on the pathophysiology of conditions that may otherwise present as isolated proteinuria or steroid-resistant nephrotic syndrome.^3^^,^^4^ Kidney biopsies in these cases typically show pathological features such as focal segmental glomerulosclerosis, minimal change disease, or diffuse mesangial sclerosis.^5^

As a result, recent guidelines recommend genetic screening for patients with steroid-resistant nephrotic syndrome,^6^ or atypical features such as associated extrarenal manifestations^3^ or the absence of edema.^7^^,^^8^ This shift toward genetic testing allows for more personalized treatment strategies for pediatric patients with complex, multisystemic diseases associated with proteinuria.

ReNU syndrome is a very recently described genetic condition caused by heterozygous variants in the *RNU4-2* gene, which encodes the U4 small nuclear RNA, a key component of the major spliceosome.^9^^,^^10^ This syndrome is characterized by neurological and neurodevelopmental features, including intellectual disability, microcephaly, short stature, hypotonia, seizures, and motor delay. Kidney involvement has also been reported, mostly in the form of congenital abnormalities of the kidney and urinary tract.^10^ However, to date, isolated proteinuria has not been described in patients with ReNU syndrome.

Here, we presented a new case of ReNU syndrome with kidney involvement, specifically characterized by isolated proteinuria, thereby expanding the clinical spectrum of this condition.

## Case Report

**Figure 1:**
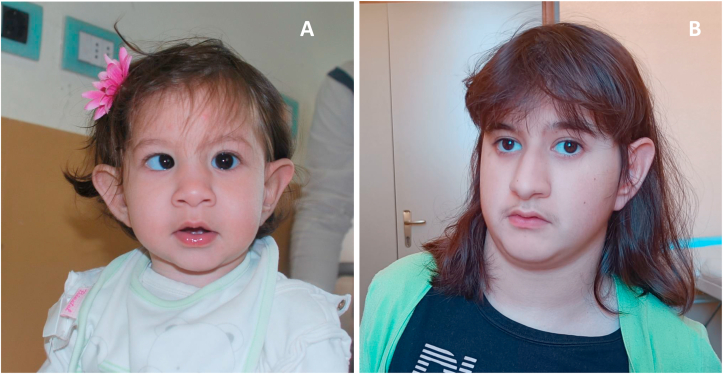
Facial features of the reported patient with ReNU syndrome during her first months of life (A) and at the latest follow-up at 16 years of age (B).

A 3-month-old girl was referred to our center for persistent proteinuria, with occasional microhematuria, despite having normal kidney function. The prenatal history was notable for a small head circumference at prenatal ultrasound. Fetal brain magnetic resonance imaging showed reduced brain hemispheres and mild enlargement of the peri-encephalic spaces in the posterior parietal region. At the referral, her weight and length were below the 3rd percentile for age and sex, and the child exhibited strabismus and dysmorphic features, including epicanthus, a depressed nasal bridge, and low-set, large ears (Fig 1A).

**Figure 2:**
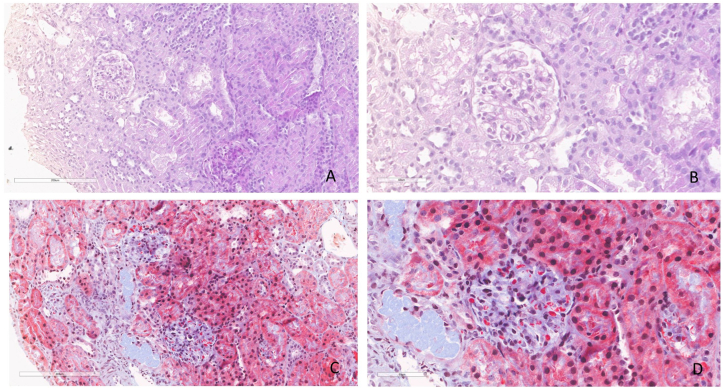
Histological sections of kidney tissue showing minimal change disease. (A) PAS stain, 20×; (B) PAS stain, 40×; (C) Trichrome stain, 20×; (D) Trichrome stain, 40×. Glomeruli show preserved capillary wall thickness, a nonexpanded mesangium, and the absence of deposits, consistent with minimal change disease. The tubular compartment is within normal limits. Abbreviations: PAS, periodic acid-Schiff.

Over the following months, the patient continued to exhibit persistent, nephrotic-range, glomerular proteinuria (spot urinary protein-to-creatinine ratio of 2.8-3.3 mg/mg) and severe albuminuria, without microhematuria. Kidney function remained normal (serum creatinine level of 0.18 mg/dL), and no other biochemical abnormalities were noted (cholesterol, triglycerides, and immunoglobulin levels were normal*)*. Kidney ultrasound was unremarkable, and edema was never observed. A kidney biopsy, performed at 15 months of age, showed minimal change disease on light microscopy (Fig 2A-D), with slight podocyte effacement observed on the ultrastructural evaluation. No other abnormalities were found. Given the persistence of asymptomatic nephrotic range proteinuria (urinary protein-to-creatinine ratio of 2.2 mg/mg), a trial with oral prednisone was conducted at 23 months, but did not yield any improvement. Therefore, antiproteinuric therapy was started, initially with angiotensin-converting enzyme inhibitors, later combined with angiotensin II receptor blockers for the persistence of nephrotic-range proteinuria. At the most recent follow-up in July 2025, at 16 years of age, proteinuria persisted at a nephrotic range with combined angiotensin-converting enzyme inhibitor/angiotensin II receptor blocker therapy (urinary protein-to-creatinine ratio of 2.5 mg/mg), and kidney function remained normal (serum creatinine level of 0.51 mg/dL). No microhematuria or other abnormalities were detected.

During the follow-up, additional features, suggesting an underlying congenital syndrome (dysmorphic features [Fig 1B], autistic traits, intellectual disability, and hypothyroidism), were noted, prompting genetic testing. Whole-genome sequencing, next-generation sequencing for focal segmental glomerulosclerosis, comparative genomic hybridization-array, and whole-exome sequencing initially yielded no pathogenic results. A repeated genetic analysis using whole-genome sequencing-trio, performed in September 2024, identified a de novo heterozygous variant in the *RNU4-2* gene (chr12(GRCh38):g.120291839_120291840 (NR_003137.2):n.64_65insT), exon 1. This variant was classified as pathogenic according to the American College of Medical Genetics and Genomics guidelines and is the most common mutation associated with ReNU syndrome. Notably, mutations in the RNU4-2 gene were not previously recognized as pathogenic before the description of ReNu syndrome.^9^^,^^10^ The variant was absent in both parents, confirming its de novo origin. The patient’s clinical presentation is consistent with the diagnosis.

## Discussion

To our knowledge, this is the first report of a patient with isolated persistent proteinuria as the sole kidney manifestation in the context of ReNU syndrome.

ReNU syndrome is a newly described entity, characterized by hypotonia, intellectual disability, autism spectrum disorder, growth restriction, microcephaly, and multisystemic involvement, including ocular (strabismus), endocrinological (hypothyroidism), cardiovascular, gastrointestinal, cutaneous, skeletal, dental, and genitourinary features.^9^^,^^10^ To date, 189 cases have been described. The de novo heterozygous variant in the *RNU4-2* gene (n.64_65insT) identified in our patient is classified as pathogenic and associated with ReNU syndrome. The clinical presentation of the proband is consistent with this diagnosis.

The kidney phenotype in our patient is characterized by isolated persistent proteinuria, with normal kidney function, no anatomical abnormalities of the kidney and urinary tract, and histological features consistent with minimal change disease on kidney biopsy. A comprehensive nephrological work-up, including laboratory investigations, imaging, kidney biopsy, and broad genetic testing, ruled out other known genetic or acquired causes of proteinuria. This supports the conclusion that the proteinuria observed in our patient is directly related to ReNU syndrome, rather than being coincidental or secondary to another condition.

This presentation is distinct from the kidney manifestations previously reported in ReNU syndrome, which typically include recurrent urinary tract infections, kidney dysplasia, bilateral pelvicalyceal dilation, vesicoureteral reflux, nephrocalcinosis, and kidney stones.^10^

*RNU4-2* encodes the U4 small nuclear RNA, a critical component of the spliceosome. Mutations in the *RNU4-2* gene, such as those described in ReNU syndrome, likely disrupt the splicing process, leading to abnormal protein production or the complete absence of proteins essential for various pathways. This helps to explain the multisystemic features of ReNU syndrome, including the neurological and kidney involvement. We propose that proteinuria in patients with ReNU syndrome may result from functional defects in podocytes or other components of the glomerular filtration barrier.

Identifying the presence of proteinuria in patients with ReNU syndrome is of paramount importance because proteinuria is a well-established early biomarker for progressive kidney dysfunction. Several existing and emerging antiproteinuric agents can slow the decline in glomerular filtration rate in patients with proteinuric nephropathies.^11^, ^12^, ^13^

Pediatric patients with isolated nephrotic-range proteinuria often show a low rate of response to steroids and are defined as having steroid-resistant nephrotic syndrome when treated according to guidelines for idiopathic nephrotic syndrome.^7^ Several gene mutations have been associated with steroid-resistant nephrotic syndrome or focal segmental glomerulosclerosis.^3^^,^^5^ Most of these mutations affect genes encoding for proteins of the glomerular filtration barrier, although other genes, including mitochondrial genes, have also been implicated. The likelihood of a genetic etiology increases in the presence of atypical features, such as absence of edema^7^ or extrarenal manifestations.^3^ Accurate genetic diagnosis can help avoid unnecessary immunosuppressive therapy, identify related clinical features, and enable family screening.

Despite the continuous advancement in genetic testing, some patients with a likely genetic basis for proteinuria remain undiagnosed. Whole-genome sequencing is the most comprehensive genetic test available and can provide an accurate diagnosis, although the potential for false positives, the complexity of the reading, and its costs limit its widespread application.

To our knowledge, this is the first case of isolated, persistent proteinuria associated with ReNU syndrome. Based on the findings from this case, we proposed that proteinuria is a previously undocumented additional feature of ReNU syndrome, thereby expanding the phenotypic spectrum of this new condition. We recommend testing for *RNU4-2* variants in patients with unexplained proteinuria and syndromic features. Additionally, we suggest that patients with ReNU syndrome be routinely monitored for the presence of proteinuria, even in the absence of edema or other signs of hypoalbuminemia. This approach could help clarify the true prevalence and natural history of proteinuria in patients with ReNU syndrome and allow early, targeted intervention to prevent the progression of kidney damage. If the association with pathogenic *RNU4-2* variants and proteinuria is confirmed, *RNU4-2* should be included in genetic panels for patients presenting with proteinuria.
